# A rare case of morphea following zilucoplan treatment for generalized myasthenia gravis

**DOI:** 10.1016/j.jdcr.2026.06.066

**Published:** 2026-07-10

**Authors:** Sydney Kenney, Alec Hou, Samuel Hwang

**Affiliations:** a Kaiser Permanente Bernard J. Tyson School of Medicine, Pasadena, California; b Department of Dermatology, University of California, Davis Health System, Sacramento, California

**Keywords:** drug-induced morphea, localized scleroderma, zilucoplan

## Introduction

Morphea (localized scleroderma) is a chronic inflammatory disorder characterized by excessive collagen deposition in the skin and sometimes underlying tissues, leading to fibrosis and variable clinical sequelae. The pathophysiology involves a complex interplay of genetic predisposition, immune dysregulation, and environmental triggers. Key mechanisms include vascular dysfunction, T helper cell imbalances, and upregulation of profibrotic cytokines which drive fibroblast activation and extracellular matrix production.^1^^,^^2^ Complement involvement in the development of morphea is not well understood, however, the classical pathway has been implicated in endothelial cell injury and subsequent immune activation, preceding profibrotic processes. Complement components such as complement 3, 4, and 5 (C3, C4, and C5) contribute to recruitment of inflammatory cells and perpetuation of tissue damage thought to interact with T helper cell pathways, especially Th1 and Th17 in early disease, and Th2 in later fibrotic stages, leading to increased production of profibrotic cytokines and excessive collagen deposition in localized scleroderma.^3^^,^^4^

Clinically, morphea presents as indurated, sclerotic plaques with violaceous borders and central hypo- or hyperpigmentation. Subtypes include plaque-type, generalized, linear, and deep morphea, with linear and deep forms potentially causing significant functional impairment due to involvement of underlying fascia, muscle, or bone.^2^ Early lesions are erythematous and edematous, progressing to firm, ivory-colored plaques with potential atrophy and pigmentary changes. Systemic symptoms and internal organ involvement are rare, distinguishing morphea from systemic sclerosis.^1^^,^^3^ We present a rare patient case of developing localized scleroderma after commencing a C5 complement inhibitor, zilucoplan, for the treatment of generalized myasthenia gravis.^5^

## Case presentation

Our patient is a 46-year-old male with a past medical history of thymoma (status post-thymectomy) and acetylcholine receptor antibody positive generalized myasthenia gravis (AChR+ gMG). He was prescribed daily subcutaneous zilucoplan (32.4 mg/0.81 mL, 0.3 mg/kg) in combination with mycophenolic acid. Mycophenolic acid was discontinued after the development of condyloma acuminata; therefore, his sole therapy for gMG was zilucoplan. Six months later, he presented to dermatology clinic for evaluation of new injection-site skin changes. Initial exam revealed pink-orange, palpable, indurated plaques scattered over the lower abdomen, bilateral anterior thighs (Fig 1, *A*-*C*), and left forearm (Fig 2).

**Fig 1:**
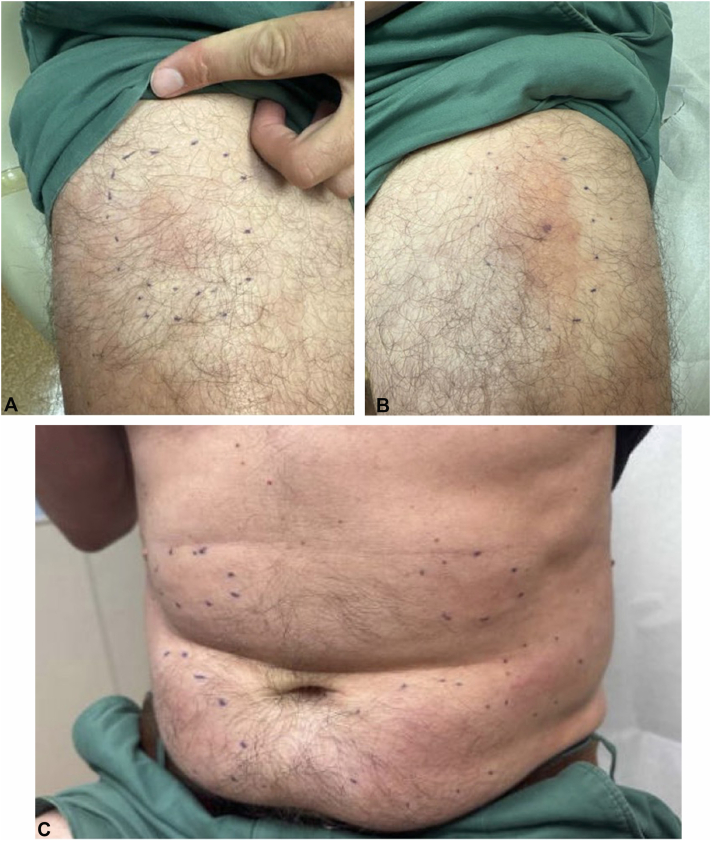
Clinical photos at initial presentation. Bilateral anterior thighs displayed pink, firm morphea plaques encompassed in surgical marking pen **(A** and **B)**. Right thigh lesion with evident injection site ecchymosis in center of sclerotic plaque **(B)**. Five separate scattered pink, poorly demarcated plaques across the abdomen located around various injection sites **(C)**.

**Fig 2:**
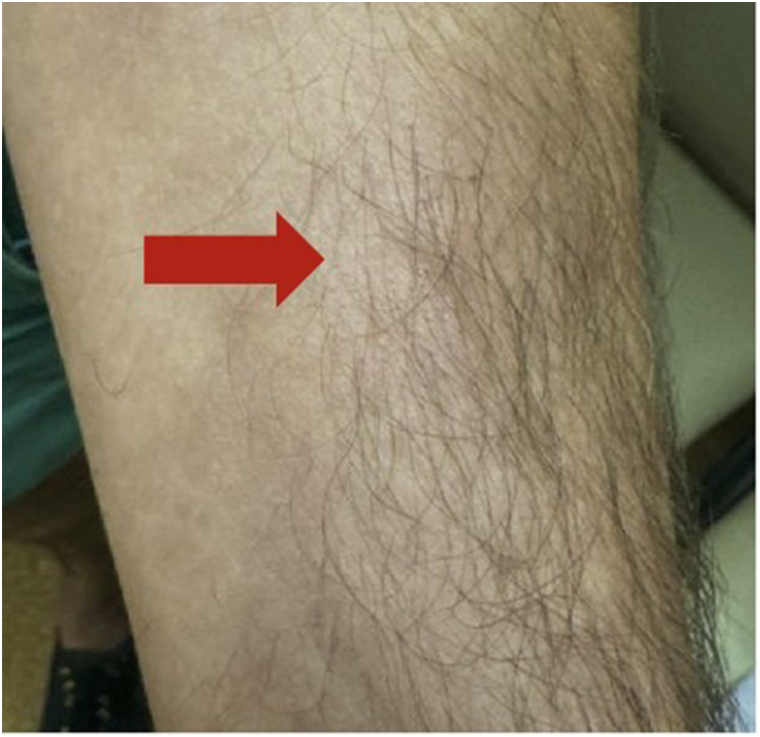
Clinical photo of left ventral forearm with morphea. Faint pink-orange palpable indurated morphea plaque on the left forearm where the patient denies injecting medication or any recent or past trauma. *Red arrow* to aid identifying a faint plaque.

The differential diagnosis included injection site reactions, morphea, systemic sclerosis, cutaneous sarcoidosis, cutaneous lymphoma, and cutaneous or systemic tuberculosis. Comprehensive metabolic panel, complete blood count, tuberculosis testing, anti-RNP, anti-SCl70, and anti-centromere antibodies were all unremarkable, making systemic sclerosis and tuberculosis unlikely. Punch biopsy of the anterior thigh lesion revealed epidermal atrophy, thick dermal collagen bundles extending into the subdermal layer, and moderate lymphocytic infiltrate, more suggestive of morphea (Fig 3, *A*-*C*). The absence of epidermotropism features and granulomas ruled down cutaneous sarcoidosis and lymphomas, respectively. Zilucoplan-related morphea was suspected and he began topical clobetasol ointment twice a day and agreed to restrict injections to the anterior thighs, avoiding the abdomen. The patient also started phototherapy treatment thrice weekly and received a series of intralesional triamcinolone injections to the most symptomatic thigh lesions. On follow-up examination 2 months later, the morphea plaques, including the isolated forearm lesion, demonstrated significant improvement, with reduced erythema, as well as decreased firmness upon palpation. The patient ultimately ceased usage of zilucoplan altogether, leading to resolution of all skin lesions on examination 6 months after discontinuation.

**Fig 3:**
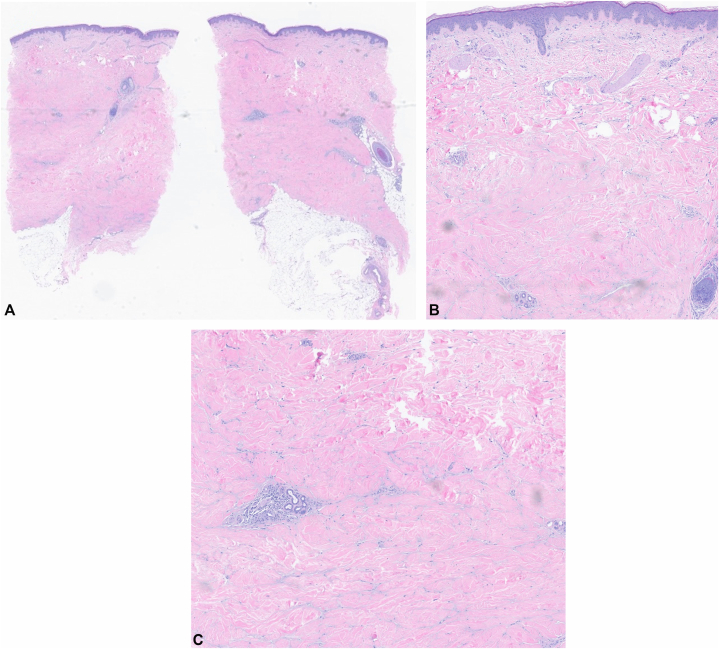
Histopathology of punch biopsy on the left thigh, H&E 10× **(A)**, 40× **(B** and **C)** Rectangular biopsy illustrates epidermal atrophy, dense collagen bundles in the dermis extending into the subcutaneous layer with sparse lymphocytic infiltrates and present plasma cells, consistent with localized scleroderma.

## Discussion

The pathophysiology of gMG includes autoantibody-mediated complement activation and subsequent degradation of the neuromuscular junction, thus worsening disease burden. Complement C5 inhibitors such as eculizumab and zilucoplan may be used in combination with standard gMG treatments to address this disturbance.^4^^,^^6^ Drug-induced morphea has been associated with medications such as injectable interferon gamma (IFNγ), bleomycin, beta receptor antagonists, vitamin analogs including B12 and K, and vaccinations such as corona virus 19 (COVID-19).^6^^,^^7^ Morphea development at injection sites occurred in 10 of 213 patients enrolled in open-label extension studies of subcutaneous zilucoplan. While such adverse cutaneous events with complement inhibitors have been rarely described, the clustering of morphea cases in the extension studies strengthens the suspicion of a true drug-induced phenomenon rather than an isolated coincidence in our patient’s case.^8^ However, the presence of morphea in a location that had never been injected with zilucoplan may suggest that higher systemic doses may cause sclerosis in indiscriminate locations possibly prone to autoimmune tissue disease. Ultimately, the numerous sclerotic plaques resolved 6 months after complete discontinuation of zilucoplan by our patient.

There are no proposed mechanisms for morphea development in complement inhibition settings in current literature. However, dysregulation of the classical complement pathway may provide the framework for hypothesizing the relationship between complement blockade and aberrant fibroblast activation. Complement plays complex roles in immune tolerance and clearance of apoptotic cells; its inhibition could theoretically impair these protective functions and unmask or trigger autoimmune responses in genetically predisposed individuals. While zilucoplan inhibits C5 cleavage and prevents formation of C5a and the membrane attack complex, inhibition could paradoxically alter immune homeostasis. Furthermore, the pathogenesis of radiation-induced morphea has been thought to systemically prime fibroblasts with aberrant Myc gene activation and elevated osteopontin, rather than solely injuring targeted tissue.^9^ In our patient, the underlying immune dysregulation from gMG may have primed fibroblasts systemically, explaining the formation of a morphea plaque distant from zilucoplan injections. Therefore, complement components implicated in fibrosis and wound-healing pathways combined with autoimmune dysregulation of fibroblasts in gMG could initiate profibrotic cascades leading to sclerosis in susceptible patients.^5^^,^^6^^,^^9^^,^^10^

This case expands the spectrum of drug-related morphea as a possible rare cutaneous side effect of a complement-targeting therapy of generalized myasthenia gravis. Since zilucoplan is in advanced clinical trials for several neuromuscular and hematologic diseases, awareness among dermatologists and prescribing physicians is warranted. Early recognition of morphea-like changes and prompt discontinuation of therapy, alongside standard treatments such as corticosteroids and phototherapy, may prevent progression and irreversible fibrosis.

## Conflicts of interest

None disclosed.
